# Exploring multiple effects of Zn_0.15_Mg_0.85_O nanoparticles on *Bacillus subtilis* and macrophages

**DOI:** 10.1038/s41598-018-30719-9

**Published:** 2018-08-16

**Authors:** Sandrine Auger, Céline Henry, Christine Péchoux, Sneha Suman, Nathalie Lejal, Nicolas Bertho, Thibaut Larcher, Slavica Stankic, Jasmina Vidic

**Affiliations:** 1 Micalis Institute, INRA, AgroParisTech, Université Paris-Saclay, 78350 Jouy-en-Josas, France; 2 Micalis Institute, PAPPSO, INRA, AgroParisTech, Université Paris-Saclay, 78350 Jouy-en-Josas, France; 3 Université Paris-Saclay, Génétique Animale et Biologie Intégrative, UMR1313, INRA, 78350, Jouy-en-Josas, France; 4 Sorbonne Université, UPMC Paris 06, CNRS-UMR 7588, Institut des NanoSciences de Paris, 7500, Paris, France; 5 Department of Chemical and Biomolecular Engineering, Whiting School of Engineering, Johns Hopkins University, Baltimore, USA; 6 Université Paris-Saclay, Virologie et Immunologie Moléculaires, UR892, INRA, 78350, Jouy-en-Josas, France; 7 INRA, UMR0703 APEX, Oniris, 44000, Nantes, France

## Abstract

The increasing number of multidrug resistant bacteria raises a serious public-health concern, which is exacerbated by the lack of new antibiotics. Metal oxide nanoparticles are already applied as an antibacterial additive in various products used in everyday life but their modes of action have remained unclear. Moreover, their potential negative effects to human health are still under evaluation. We explored effects of mixed metal oxide Zn_0.15_Mg_0.85_O on *Bacillus subtilis*, as a model bacterial organism, and on murine macrophages. Zn_0.15_Mg_0.85_O killed planktonic bacterial cells and prevented biofilm formation by causing membrane damages, oxidative stress and metal ions release. When exposed to a sub-inhibitory amount of Zn_0.15_Mg_0.85_O, *B*. *subtilis* up-regulates proteins involved in metal ions export, oxidative stress response and maintain of redox homeostasis. Moreover, expression profiles of proteins associated with information processing, metabolism, cell envelope and cell division were prominently changed. Multimode of action of Zn_0.15_Mg_0.85_O suggests that no single strategy may provide bacterial resistance. Macrophages tolerated Zn_0.15_Mg_0.85_O to some extend by both the primary phagocytosis of nanoparticles and the secondary phagocytosis of damaged cells. Bacterial co-treatment with ciprofloxacin and non-toxic amount of Zn_0.15_Mg_0.85_O increased antibiotic activity towards *B*. *subtilis* and *E*. *coli*.

## Introduction

Providing an efficient and safe treatment for bacterial multi-drug resistant strains is a major health challenge worldwide^1^. Some bacterial strains have the potential to adhere on any surfaces and form slimy layer known as a biofilm. The formation of biofilms enhances the bacterial resistance to current treatments by slowing penetration of the antibiotic into the biofilm, altering chemical microenvironment of bacterial cells and by enabling cell differentiation similar to spore formation^2^. There is an urgent need to develop novel pharmacological approaches to fight multidrug-resistance pathogenic bacteria and to destroy or prevent their biofilm formation or sporulation. Metal oxide nanoparticles (NP), such as ZnO, CuO and TiO_2_, have already been proven as a good candidate to fight various bacteria^3–9^. However, their therapeutic applications as antibacterial agents are still limited as these metal oxides at nanoscale may exhibit high cytotoxicity on mammalian cells^10^. Thus, new insights into the complex tri-part interactions, bacterial cells-metal oxide nanoparticles-mammalian cells, are required to rationally design novel biocompatible antibacterial agents. We hypothesis here that mixed metal oxide NPs with synergic effects of two oxides may provide a new solution for an infectious disease treatment.

MgO NP is a commonly used model system for studying surface reactions at nanoscale, mainly due to its simple rock-salt crystal structure and purely inorganic nature. Unlike other NPs with antibacterial activity, such as colloidal silver NPs, which release cytotoxic Ag^+^ ions, or photocatalytic nanoparticles that demand intense irradiation to be efficient, nanostructured MgO are low cost, easy to manipulate and show intrinsic biocompatibility. Two strategies have been proposed to improve the antibacterial activity of MgO NPs^11,12^. First, antibacterial efficiency of MgO can be significantly enhanced by decreasing the size of MgO nanocubes to less than 10 nm^13^. The lowering the particles’ size facilitates NP penetration into bacterial cells and biofilms and results in high crystal surface area which enhances particles’ reactivity^14^. Second, the admixing of another cation into the MgO NPs may significantly improve particle’s antibacterial efficiency^11^. Doping MgO with Ca, Zn or Li was shown to modify MgO morphology, surface structure and physicochemical properties^15–17^. For instance, while MgO nanocubes only partially inhibited bacterial growth, mixed biphasic ZnMgO NPs, of a similar size, showed a high antibacterial efficiency^12^.

Herein, we used small monophasic MgO NPs decorated at edges with Zn (15 at. %), called Zn_0.15_Mg_0.85_O^18^. Zn_0.15_Mg_0.85_O were regular cubic NPs of a narrow particle size and shape distribution with a high surface specific area and a large number of low-coordinated ions and/or surface defects^11,19^. In consequence, Zn_0.15_Mg_0.85_O NPs are expected to show enhanced surface reactivity and, thus, a high antibacterial efficiency.

*Bacillus subtilis* was used as a model bacterial specie to evaluate Zn_0.15_Mg_0.85_O NP effects on planktonic bacterial cells, biofilms and spores. In depth, the NP size, interfacial potential and ROS production in solutions, antibacterial efficiency as well as effects on cellular morphology and cytotoxicity were all considered. The viability and NP intake by mammalian cells were studied in macrophage cells exposed to Zn_0.15_Mg_0.85_O at various concentrations. Macrophages were chosen as they exert scavenger’s role to eliminate pathogens and harmful particles from the body. Taking together, we expect that this study sheds light on molecular mechanisms involved in metal oxide interactions with bacteria and immunological cells and reveals potentials for improving design of doped metal oxide NPs for biomedical applications.

## Results

### Zn_0.15_Mg_0.85_O nanoparticles characterization in solution

Zn_0.15_Mg_0.__85_O were produced as monophasic nanocubes with a 4 nm average size (Fig. S-1). The low Zn/Mg ratio was chosen to assure single crystal structure of doped MgO nanocubes and to prevent enhanced cytotoxicity. Indeed, ZnO nanomaterials are instable in aqueous solutions and have tendency to release highly toxic Zn^2+^ ions^5,20^.

**Figure 1:**
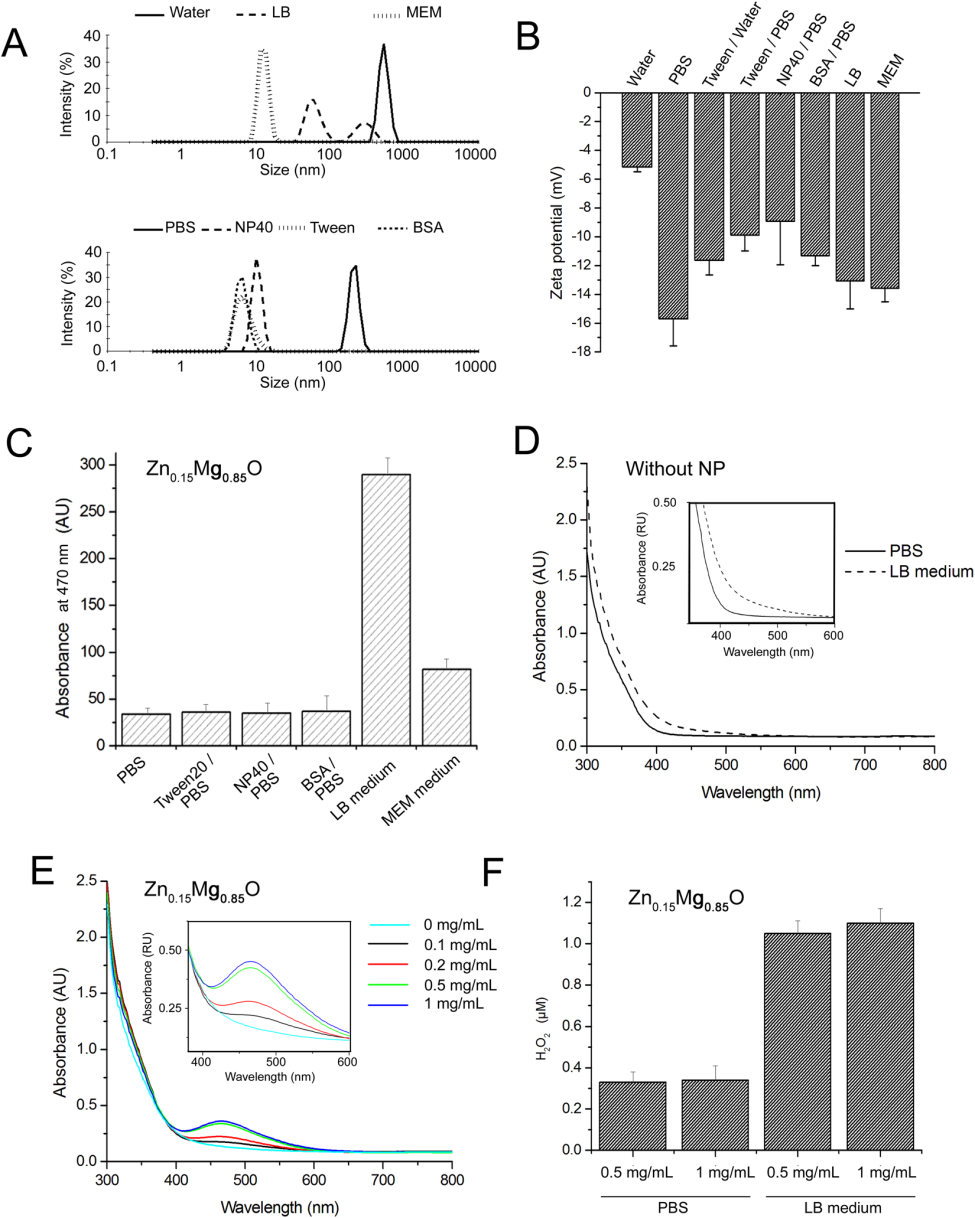
Characterization of Zn_0.15_Mg_0.85_O in solutions. (**A**) DLS measurements showing size distributions of 0.1 mg/mL Zn_0.15_Mg_0.85_O in pure water, bacterial LB medium, mammalian cell medium (MEM), in PBS, pH 7.4, and PBS containing 0.05% NP40, 0.05% Tween-20 or 2 mg/mL BSA. All solutions were incubated for 24 h at room temperature before measurements. (**B**) Zeta–potential analysis of Zn_0.15_Mg_0.85_O NPs in different solutions used in this work; (**C**) O_2_^•−^ generation of Zn_0.15_Mg_0.85_O NPs (0.1 mg/mL, initial concentration) in various solutions, at 25 °C obtained by measuring reduction of 200 µM XTT; (**D**) No reduction of 200 µM XTT was observed in control experiments without NPs. (**E**) Generation of O_2_^•−^ by different concentrations of NPs in PBS; (**F**) Production of H_2_O_2_ by Zn_0.15_Mg_0.85_O NPs in PBS and LB obtained by the Amplex red assay.

Zn_0.15_Mg_0.85_O NPs tended to aggregate and precipitate in water. To check whether the solubility of Zn_0.15_Mg_0.85_O NPs increased when ions, detergent or proteins were added to the water, DLS analysis was performed to measure the mean hydrodynamic radius (R_H_) of NPs in aqueous solutions (Fig. 1A). The R_H_ value of Zn_0.15_Mg_0.85_O were found to shift from about 1 µm in water to 0.1 µm in PBS containing 150 mM NaCl. Such sizes were much higher than the primary nanoparticle size indicating aggregation of particles in both water and PBS. However, the possibility that NPs were dissolved to some extend cannot be roll out. DLS cannot detect small particles in the presence of large ones as the scattered light at smaller particle sizes is extremely reduced compared with that at the larger particles^21^. The apparent smaller particles with R_H_ ~ 10 nm were found when Zn_0.15_Mg_0.85_O was dissolved in water solution containing BSA, Tween20 or NP40 indicating that protein and non-ionic detergents increased solubility of Zn_0.15_Mg_0.85_O NPs (Fig. 1A). This is in line with the well-established finding that surfactants adsorb on metal oxide NPs and stabilize them in solutions^11,22^. Two main populations of NP aggregates with R_H_ of about 50 and 500 nm were found for Zn_0.15_Mg_0.85_O admixed with LB bacterial medium, while only a single peak with R_H_ ~10 nm was observed for Zn_0.15_Mg_0.85_O added to mammalian cell culture medium (MEM, completed with 10% serum). The dissolution of Zn_0.15_Mg_0.85_O NPs in MEM was probably enhanced by BSA from serum.

To verify whether interfacial potential of Zn_0.15_Mg_0.85_O NPs differed in various solutions, zeta potential of dissolved particles was measured. Negative zeta potential was obtained in all solution tested (Fig. 1B). The lowest zeta potential magnitude was found for Zn_0.15_Mg_0.85_O in ultrapure water (−5.16 ± 0.34 mV) confirming the particles aggregation in water.

### Zn_0.15_Mg_0.85_O nanoparticles generate ROS

Antibacterial activity of metal oxide NPs is thought to be mediated by reactive oxygen species (ROS)^23^. To verify whether Zn_0.15_Mg_0.85_O produced O_2_^*−^ in aqueous solutions, the XTT assay was performed. When reduced by O_2_^*−^ XTT forms water soluble XTT-formazan which adsorbs light at 470 nm. Figure 1C shows the increase in XTT absorption peak intensity obtained in various solutions containing Zn_0.15_Mg_0.85_O. Upon 5 h incubation, 0.5 mg/mL of Zn_0.15_Mg_0.85_O generated O_2_^*−^ in all solutions tested while no absorption peak was observed in the absence of NPs (Fig. 1D). The highest concentration of O_2_^*−^ was detected in LB medium. The concentration of O_2_^*−^ increased with increasing Zn_0.15_Mg_0.85_O concentration and reached saturation at 1 mg/mL (Fig. 1E).

To check whether Zn_0.15_Mg_0.85_O NPs can generate H_2_O_2_ without UV illumination, the Amplex red assay was performed (Fig. 1F). The concentration of H_2_O_2_ in solutions was calculated using a calibration curve obtained with pure H_2_O_2_. Zn_0.15_Mg_0.85_O NPs at 0.5 mg/mL generated (1.05 ± 0.07) µM H_2_O_2_ and (0.33 ± 0.01) µM H_2_O_2_ in LB medium and PBS, respectively over one hour. Interestingly, in both solutions, no further increase in H_2_O_2_ production was observed when NP concentration was increased from 0.5 mg/mL to 1 mg/mL (Fig. 1F).

### Antibacterial activity of Zn_0.15_Mg_0.85_O on *B*. *subtilis* planktonic cells

**Figure 2:**
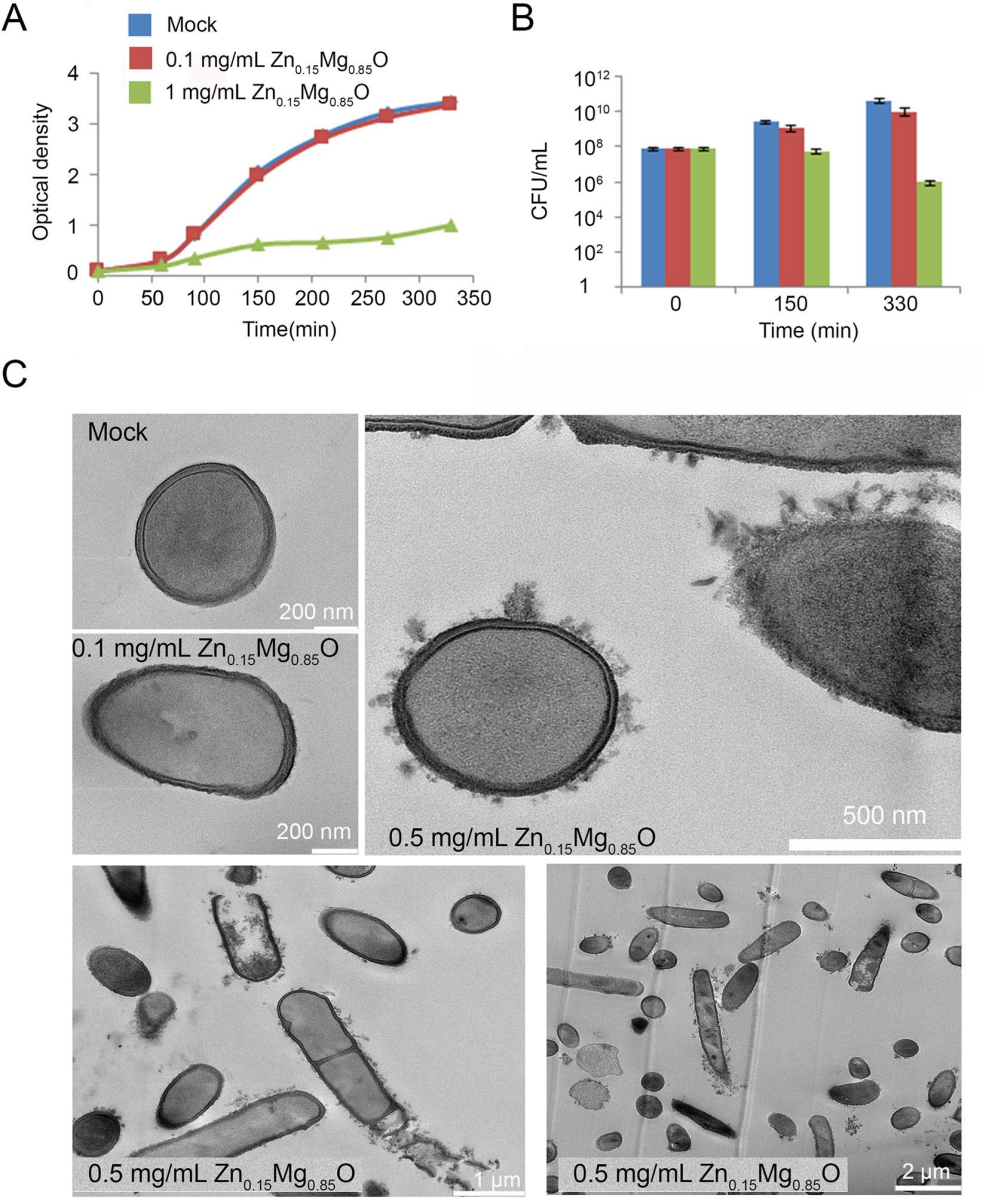
Antibacterial effects of Zn_0.15_Mg_0.85_O NPs. (**A**) Growth curves of *B*. *subtilis* in LB medium in the absence (mock) and in the presence of 0.1 mg/mL or 1 mg/mL of Zn_0.15_Mg_0.85_O nanoparticles. (**B**) *B*. *subtilis* cell viability obtained upon bacterial cell incubation with 0.1 mg/mL or 1 mg/mL Zn_0.15_Mg_0.85_O NPs in LB, as quantified by the colony counting method. (**C**) TEM cross-section observations of untreated *B*. *subtilis* (mock) or treated with 0.1 mg/mL or 0.5 mg/mL Zn_0.15_Mg_0.85_O NPs.

The antibacterial activity of Zn_0.15_Mg_0.85_O was examined against *B*. *subtilis*, a representative of Gram (+) bacteria. Growth curves of *B*. *subtilis* exposed to 0.1 or 1 mg/mL of NPs were determined by monitoring the optical density (OD) at 600 nm over time.We observed that growth of *B*. *subtilis* decreased significantly with the increasing concentration of NPs (Fig. 2A). Cell viability evaluated by the colony counting method was about 100-fold reduced as compared to the initial concentration of cells (10^8^ CFU/ml) upon incubation with 1 mg/mL Zn_0.15_Mg_0.85_O NPs after 330 min (Fig. 2B). The minimum inhibitory concentration (MIC) value of Zn_0.15_Mg_0.85_O NPs against *B*. *subtilis* evaluated by broth microdilution method with 10^4^ cfu/mL was 450 mg/L. In comparison, the MIC of Zn_0.15_Mg_0.85_O NPs against *E*. *coli* was 710 mg/L (see Supplementary information S-2 for details).

**Figure 3:**
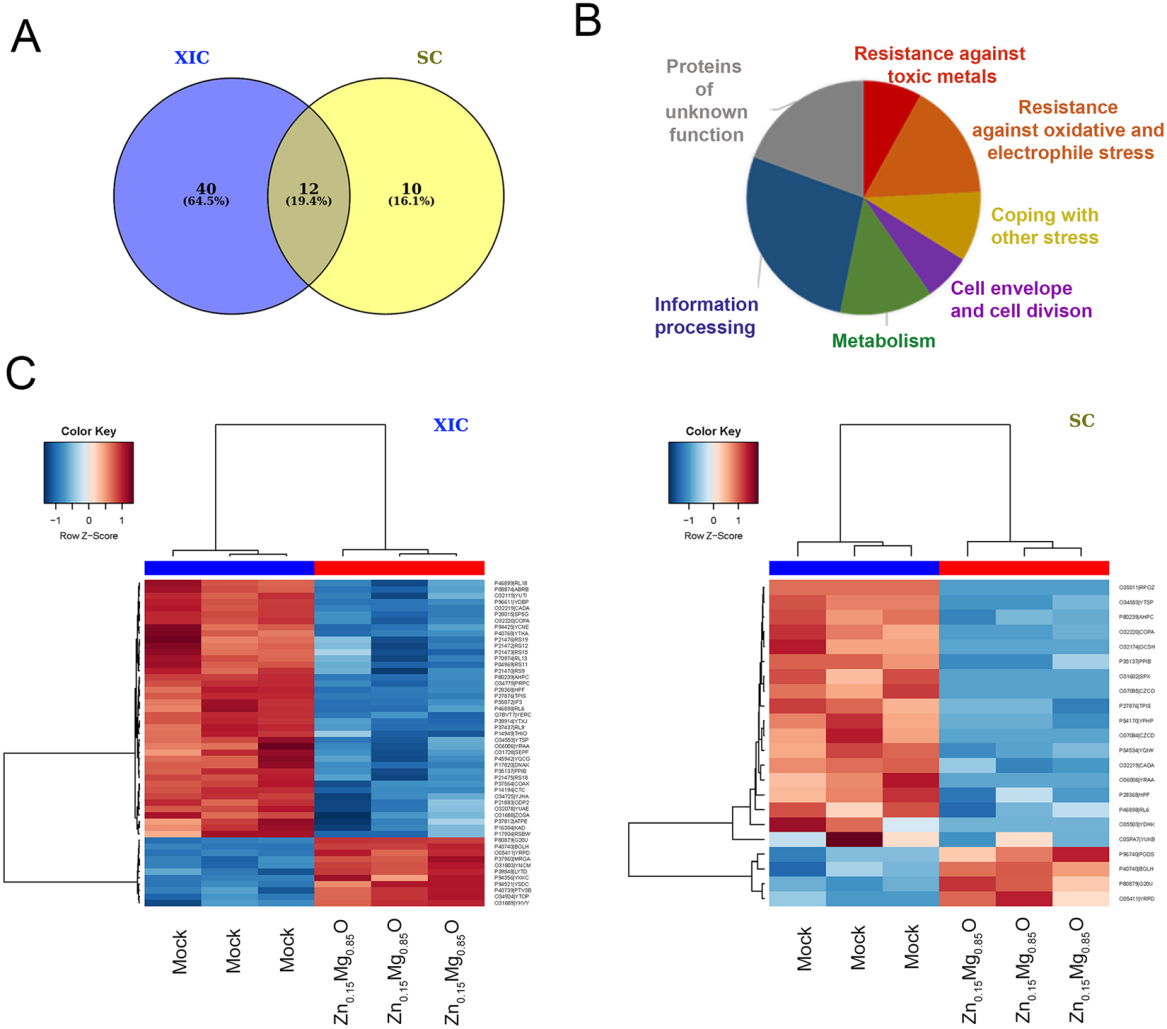
Comparative proteome analysis of *B*. *subtilis* membrane fractions of untreated bacterial cells and cells treated with 0.05 mg/mL Zn_0.15_Mg_0.85_O NPs in LB for 1 h. (**A**) Statistical analysis indicated that 62 proteins had modified abundances in treated and untreated bacteria. Two modes of label free quantification were used: spectral counting (SC) and eXtracted Ion Current ion chromatograms of each peptide (XIC). (**B**) Representation of the proteome response of *B*. *subtilis* to Zn_0.15_Mg_0.85_O NPs. The pie chart shows the size of functional categories according to the SubiWiki database. (**C**) Heatmap presentations of upregulated and downregulated proteins. Left panel, proteins quantified by XIC statistic method; right panel, proteins quantified by SC. Only proteins showing significant abundance change between untreated (mock) and treated bacteria (ANOVA, adjusted *p* value < 0.05) are displayed.

To visualize bacterial morphology upon treatment with Zn_0.15_Mg_0.85_O, TEM measurements were performed on tin cross-section of *B*. *subtilis* cells incubated with Zn_0.15_Mg_0.85_O over only 60 min. As depicted in Fig. 2C *B*. *subtilis* cultured in the absence of NPs showed intact rod-shaped and round-shaped cells protected with smooth and well-structured membrane layer. All cells were viable and no membrane damage could be observed. After exposing to sub-inhibitory concentration of Zn_0.15_Mg_0.85_O (0.1 mg/mL) bacterial cells still maintained their integrity. However, upon exposure to 0.5 mg/mL Zn_0.15_Mg_0.85_O NPs the attachment of nanoparticles to the bacteria was observed (Fig. 3C). The shape of bacterial cells changed to more irregular suggesting that Zn_0.15_Mg_0.85_O NPs injured the membrane of *B*. *subtilis*. Membrane damage further caused membrane leakage and increased cell permeability leading ultimately to bacterial death, as illustrated in Fig. 3C.

### Impact of Zn_0.15_Mg_0.85_O on *B*. *subtilis* proteome

To understand the physiological state of *B*. *subtilis* cells upon exposition to Zn_0.15_Mg_0.85_O, a comparative proteomic analysis of the membrane fractions of untreated and treated bacteria was performed. To preserve cell integrity, the bacterial culture were treated with low concentration of NPs, 0.05 mg/mL (Fig. S-3). The optimized analyses by LC-MS/MS of three biological replicates resolved more than 1550 membrane proteins or proteins attached to the membrane (Table S1). Among them, 62 proteins showed significant abundance variations (p < 0.05; Kruskal-Wallis test and one way ANOVA) in response to Zn_0.15_Mg_0.85_O NPs (Fig. 3A). Identified proteins mainly belong to 7 large functional categories according to their annotation in the SubtiWiki database^24^ (Fig. 3B).

Membrane proteins involved in toxic metal export were among the most up-regulated proteins after bacterial exposure to Zn_0.15_Mg_0.85_O NPs (Fig. 3C, Table 1). In particular, up-regulation of CzcD and CadA, that protect the cell against elevated levels of Zn^2+^-ions^25^, was observed. The presence of Zn_0.15_Mg_0.85_O NPs also induced synthesis of proteins related to redox conditions and defense against ROS as the antioxidant enzyme AhpC or bacilliredoxins BrxA and BrxB, which perform redox switch in response to oxidative stress. By contrast, we observed down-regulation of the mini-ferritins Dps and MrgA, which act as internal iron metal chelators. Proteomic analysis additionally revealed a wide and prominent effect of Zn_0.15_Mg_0.85_O NPs on proteins associated with information processing, metabolism, cell envelope dynamics or cell division (Fig. 3C; Table 2).

**Table 1:** Proteins up- or down-regulated in *B*. *subtilis* cells treated with Zn_0.15_Mg_0.85_O.

Protein name	Fold change ZnMgO vs control	Gene name	Product and Function
**Resistance against toxic metals**			
CADA	3.91	*cadA*	Cadmium export, induced by toxic metal ions (Zn(II), Cd(II), Co(II), Ni(II) and Cu(II))
CZCD	2/0	*czcD*	Cadmium, cobalt and zinc/H(+)-K(+) antiporter
CZCO	6/0	*trkA*	Cation exporter, induced in the presence of toxic metal ions (Zn(II), Cd(II), Co(II)C, Ni(II) and Cu(II))
COPA	7.61	*copA*	Copper-exporting P-type ATPase, resistance to copper
ZOSA	1.53	*pfeT*	Fe(II) efflux pump, protects the cell against iron intoxication
**Resistance against oxidative and electrophile stress**			
AHPC	3.51	*ahpC*	Alkyl hydroperoxide reductase subunit
G20U	0.28	*dps*	Mini-ferritin, iron storage protein, resistance against ethanol and paraquat
HPF	3.62	*hpf*	General stress protein, resistance against paraquat
MRGA	0.43	*mrgA*	Mini-ferritin, DNA-binding stress protein, iron storage protein
SPX	3/0	*spx*	Transcriptional regulator of many genes in response to thiol specific oxidative stress
YDBP	3.25	*ydbP*	Similar to thioredoxin
YDHK	3/0	*ydhK*	General stress protein, survival of ethanol and paraquat stresses
YPHP	9/2	*brxA*	Bacilliredoxin
YQIW	13/3	*brxB*	Bacilliredoxin
YRAA	1.54	*yraA*	General stress protein, degradation of damaged thiol-containing proteins
**Coping with other stress**			
COAX	2.32	*coaX*	Pantothenate kinase, biosynthesis of coenzyme A
DNAK	1.45	*dnaK*	Class I heat-shock protein,molecular chaperone
YKVY	0.56	*papB*	Degradation of proline-containing peptides
RSBW	1.82	*rsbW*	Anti-protein serine kinase, phosphorylates RsbV, control of SigB activity
YQCG	1.43	*yqcG*	Toxin, eliminates defective cells from developing biofilms, DNase activity
YTXJ	2.99	*ytxJ*	Unknown

**Table 2:** Proteins up- or down-regulated in *B*. *subtilis* cells treated with Zn_0.15_Mg_0.85_O.

Protein name	Fold change ZnMgO vs control	Gene name	Product and Function
**Cell envelope and cell division**			
LYTD	0.62	*lytD*	Peptidoglycan N-acetylglucosaminidase, major autolysin, cell separation
PGDS	1/4	*pgdS*	Gamma-DL-glutamyl hydrolase, polyglutamic acid degradation
SEPF	1.58	*sepF*	Part of the divisome, recruits FtsZ to the membrane
SP5G	1.63	*spoVG*	RNA-binding regulatory protein, negative effector of asymetric septation at the onset of sporulation
**Metabolism**			
ATPE	2.24	*atpE*	ATP synthase (subunit c)
BGLH	0.45	*bglH*	Phospho-beta-glucosidase, salicin utilization
GCSH	2/0	*gcvH*	Glycine cleavage system H protein for lipoic acid biosynthesis
KAD	2.03	*adk*	Adenylate kinase. ADP formation
ODP2	1.58	*pdhC*	Pyruvate dehydrogenase, links glycolysis and TCA cycle
PTV3B	0.32	*bglP*	Beta-glucoside uptake and phosphorylation, control of LicT activity
THIO	1.72	*thiO*	FAD-dependent glycine oxidase, biosynthesis of thiamine
TPIS	3.33	*tpiA*	Triose phosphate isomerase, glycolytic/ gluconeogenic enzyme
**Information processing**			
ABRB	1.71	*abrB*	Transcriptional regulator of transition state genes
PPIB	4.13	*ppiB*	Peptidyl-prolyl cis-trans isomerase (protein folding)
PRPC	1.85	*prpC*	Protein phosphatase (protein modification)
RPOZ	3/0	*rpoZ*	Omega subunit of RNA polymerase
YUKB	7/2	*yukB*	Membrane FtsK/SpoIIIE-like ATPase
CTC	3.09	*ctc*	Similar to ribosomal protein L25
IF3	1.92	*infC*	Translation initiation factor IF-3
RL6	2.08	*rplF*	50S ribosomal protein L6
RL9	1.87	*rplI*	50S ribosomal protein L9
RL13	1.77	*rplM*	50S ribosomal protein L13
RL18	1.58	*rplR*	50S ribosomal protein L18
RS11	3.02	*rpsK*	30S ribosomal protein S11
RS9	2.35	*rpsI*	30S ribosomal protein S9
RS12	2.00	*rpsL*	30S ribosomal protein S12
RS15	3.19	*rpsO*	30S ribosomal protein S15
RS18	2.63	*rpsR*	30S ribosomal protein S18
RS19	2.95	*rpsS*	30S ribosomal protein S19
**Proteins of unknown function**			
YUTI	2.56	*yutI*	Putative iron-sulfur scaffold protein
YTSP	2.57	*ytsP*	Unknown
YRPD	0.46	*yrpD*	Unknow
YUAE	1.7	*yuaE*	Unknown
YCNE	2.11	*ycnE*	Unknown
YXKC	0.64	*yxkC*	Unknown
YTKA	1.49	*ytkA*	Unknown
YJHA	1.61	*yjhA*	Unknown
YSDC	0.49	*ysdC*	Similar to endo-1.4-beta-glucanase
YTOP	0.58	*ytoP*	Similar to glutamyl aminopeptidase
YERC	1.73	*yerC*	Unknown
YNCM	0.65	*yncM*	Unknown

### Zn_0.15_Mg_0.85_O affects *B*. *subtilis* biofilm formation

**Figure 4:**
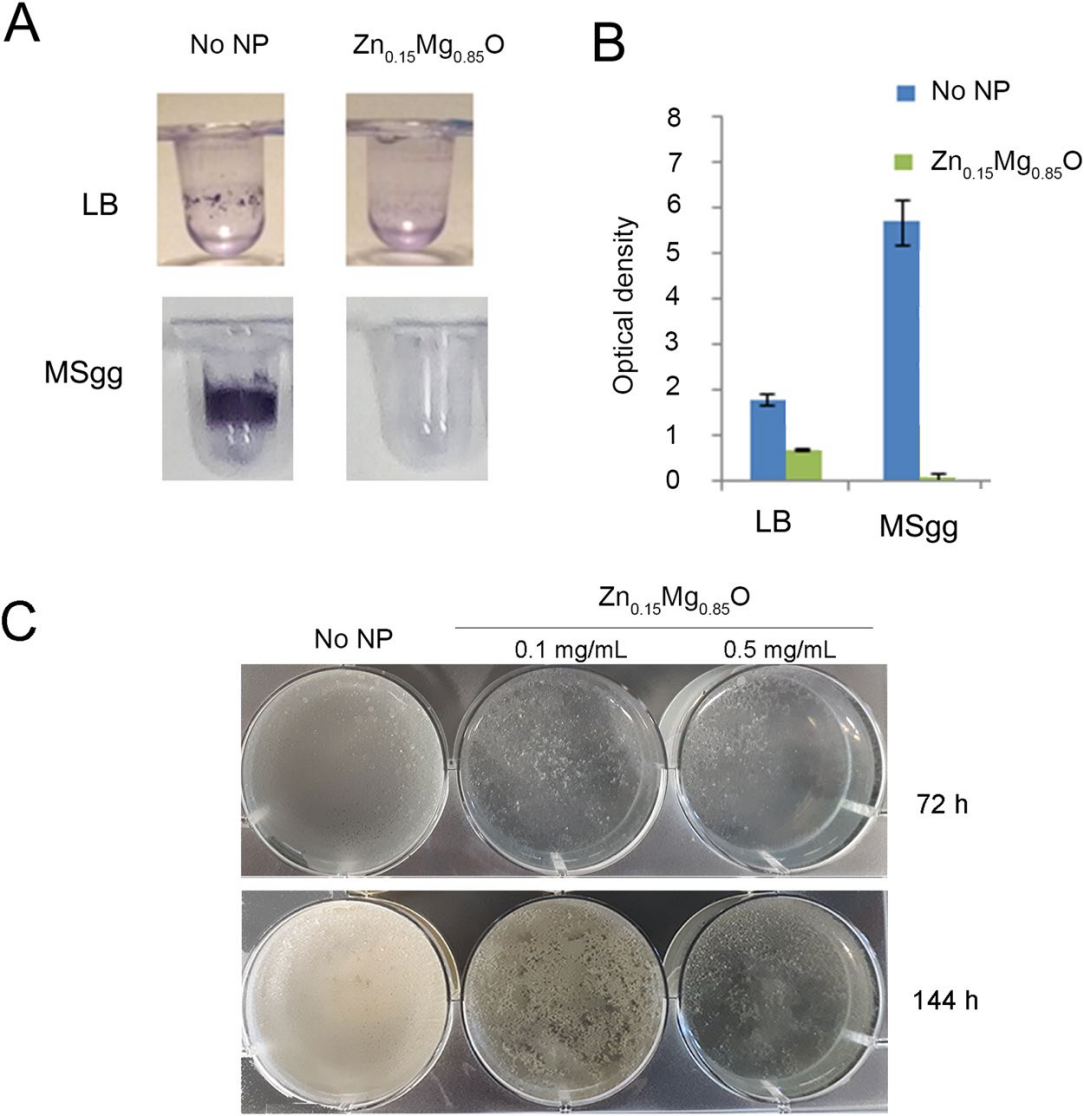
Effect of Zn_0.15_Mg_0.85_O NPs on biofilm formation by NDmed *B*. *subtilis* in LB and MSgg medium (**A,B**). Bacterial cells of were incubated in the absence of NPs and in the presence of 0.1 mg/mL Zn_0.15_Mg_0.85_O. Microtiter plates were stained with crystal violet after incubation at 30 °C without agitation for 24 h. Quantification of biofilm density was obtained by measuring OD at 595 nm of solubilized crystal violet formed in microtiter plate assay. The error bars represent mean ± SD from at least four independent experiments. (**C**) Pellicle biofilm formation by NDmed strain in MSgg medium alone and with added Zn_0.15_Mg_0.85_O NPs at 0.1 mg/mL and 0.5 mg/mL.

The ability to form biofilms is an important characteristic of many environmental bacteria, including *B*. *subtilis*. The specific structure of biofilms increases bacteria resistant to antibiotics and chemicals. The *B*. *subtilis* 168 strain and its derivatives show a reduced biofilm forming ability because of mutations accumulated during laboratory propagation^26^. For this raison, the direct effect of Zn_0.15_Mg_0.85_O NPs on biofilm formation was tested on the undomesticated strain NDmed^27^. To assess the time course of biofilm formation in the presence of NPs, microtiter plates were inoculated with NDmed strain. After 24 h of incubation, biofilms were stained with crystal violet, which stains bacterial cells and biofilm matrix components^28^ (Fig. 4A). Measurable amounts of biofilm were detected when NDmed was cultivated in the absence of NPs in both LB and biofilm-promoting MSgg medium (Fig. 4). In the presence of non-lethal concentration of Zn_0.15_Mg_0.85_O NPs (0.1 mg/mL), the biofilm was 2.7-and 10.4-fold reduced in LB and MSgg, respectively (Fig. 4A and B). Since *B*. *subtilis* forms a floating film^29,30^, the detached films might not be quantified with the crystal violet staining. We, thus, compared the dynamics of pellicle formation of NDmed biofilm in MSgg alone and in the presence of 0.1 mg/mL and 0.5 mg/mL Zn_0.15_Mg_0.85_O NPs. NDmed formed a dense robust pellicle in MSgg medium while in the presence of Zn_0.15_Mg_0.85_O NPs only thin pellicles and unstructured colonies were observed (Fig. 4C). It appears thus that Zn_0.15_Mg_0.85_O NPs have a drastic inhibitory effect on the formation of biofilms by *B*. *subtilis*.

### Zn_0.15_Mg_0.85_O did not effectively inactivate *B*. *subtilis* spores

We also examined whether Zn_0.15_Mg_0.85_O NPs destroy *B*. *subtilis* spores. Comparing, the viability of *B*. *subtilis* spores pre-incubated with 1 or 5 mg/mL NPs for 1 to 24 h to that of untreated spores by plate count experiments indicated that Zn_0.15_Mg_0.85_O had no effect on viability of *B*. *subtilis* spores. Considering the complex structure of spores, which are encased in a thick multilayered coat surrounded by the exosporium, their resistance to Zn_0.15_Mg_0.85_O NPs was expected.

### Zn_0.15_Mg_0.85_O cytotoxicity towards macrophages

**Figure 5:**
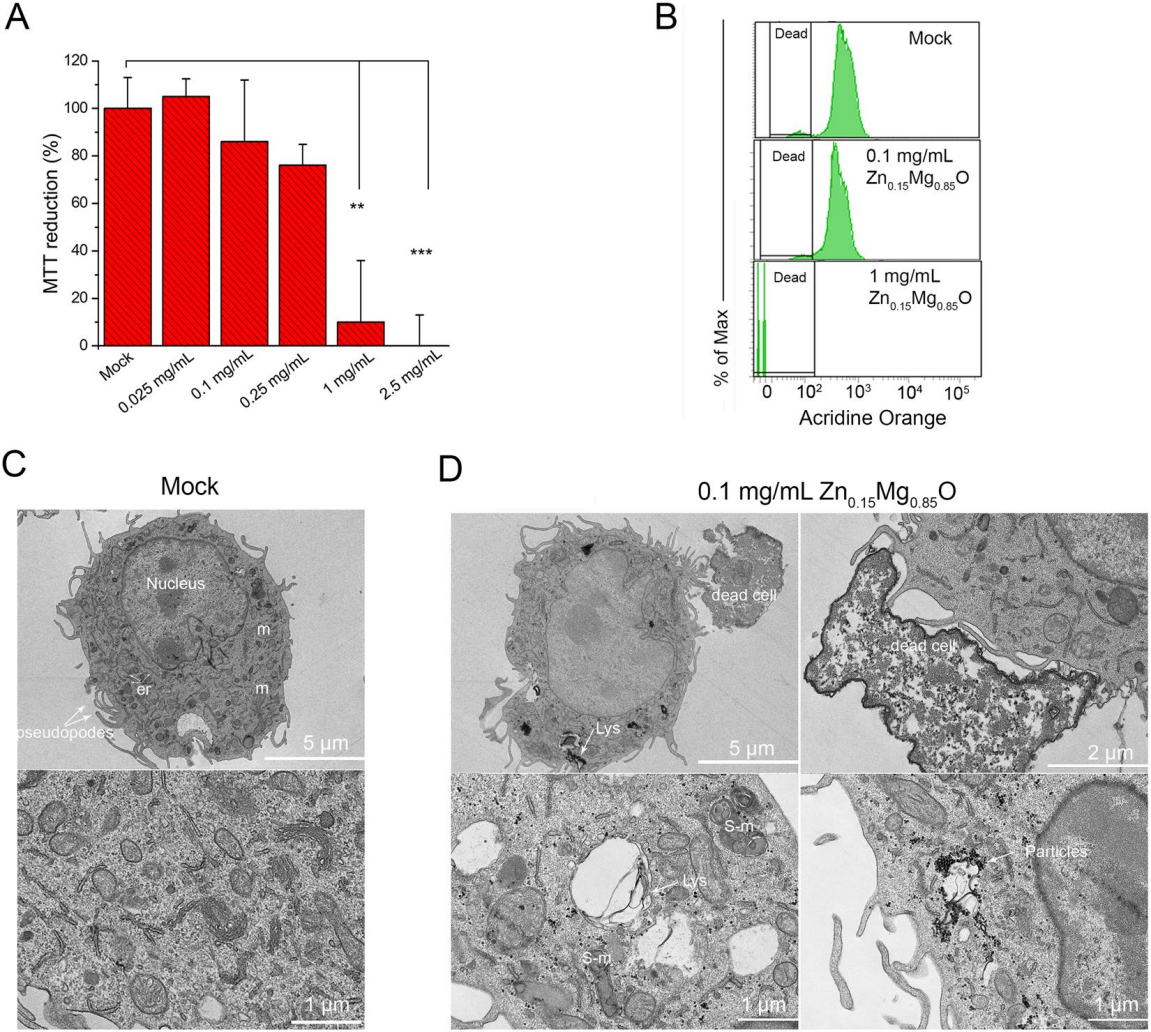
Cytotoxic effect of Zn_0.15_Mg_0.85_O NPs on macrophage cells. (**A**) MTT reduction in macrophages incubated with Zn_0.15_Mg_0.85_O NPs at various concentrations overnight. The % of MTT reduction relative to that of control cells incubated with PBS is plotted. The error bars represent SD of the means over total of 8 replicates, ** correspond to P-value < 0.01, and ***P < 0.001. (**B**) Viability of macrophages incubated with 0.1 mg/mL or 1 mg/mL Zn_0.15_Mg_0.85_O NPs overnight was estimated by acridine orange staining and flow cytometry analysis. Note that there was no significant difference in acridine orange fluorescence between untreated (mock) and cells treated with 0.1 mg/mL Zn_0.15_Mg_0.85_O. (**C**) Representative thin section electron micrographs of untreated macrophage cells and (**D**) macrophages cells incubated with 0.1 mg/mL Zn_0.15_Mg_0.85_O for 24 h. m, mitochondria; er, endoplasmic reticulum; Lys, lysosome; S-m, swelling mitochondria.

To apply a manufactured NP as an efficient and safe agent for antibacterial applications, it should specifically kill pathogenic bacteria without being toxic to mammalian cells. To test cytotoxicity of Zn_0.15_Mg_0.85_O NPs we explored their effects on the RAW 264.7 macrophage cell line. The MTT test measures the cellular reduction of the tetrazolium dye MTT as an indicator of the cell viability^31^. No significant reduction in cell viability was observed in macrophages treated with 0.025–0.25 mg/mL Zn_0.15_Mg_0.85_O NPs (Fig. 5A). In contrast, MTT reduction dropped drastically to an average of 10% to that of control cells after an exposure to 1 mg/mL Zn_0.15_Mg_0.85_O NPs; whereas MTT reduction was completed after cell treatment with 2.5 mg/mL Zn_0.15_Mg_0.85_O NPs (Fig. 5A). These data suggest that Zn_0.15_Mg_0.85_O NPs are highly cytotoxic at concentrations ≥1 mg/mL.

To verify whether Zn_0.15_Mg_0.85_O NPs damage cell membrane in macrophages, a FACS analysis was performed on treated cells stained with acridine orange (Fig. 5B). Macrophages were harvested and analyzed for necrosis after their incubation with Zn_0.15_Mg_0.85_O NPs. Acridine orange dye easily enters the cell membrane and accumulates in lysosomes. During necrosis lysosomes are ruptured due to the loss of the membrane integrity decreasing the dye fluorescence^32^. A shown in Fig. 5B no reduction of acridine orange derived fluorescence intensity was observed in cells treated with 0.1 mg/mL Zn_0.15_Mg_0.85_O NPs. However, 1 mg/mL Zn_0.15_Mg_0.85_O NPs induced 100% of cell mortality of treated macrophages. The quantitative membrane damage FACS analysis is, therefore, consistent with the cell viability test obtain with MTT assay.

TEM analysis was done on thin sections of treated macrophages to analyze effects of Zn_0.15_Mg_0.85_O NP on cellular and subcellular morphology. Untreated macrophages served as control (Fig. 5C). Ultrastructural analysis of macrophages incubated with a non-toxic dose of NP (0.1 mg/mL) revealed that cellular and organelle architecture of most treated cells changed (Fig. 5D). The electron dense areas were observed within cells suggesting that Zn_0.15_Mg_0.85_O NPs were internalized. The localization of NPs inside cells was rather dispersed showing a different degree of aggregation. Typically, electron dense aggregates were in a vicinity of membrane-rich regions. Some treated macrophages displayed features of cell death: loss of cell membrane specialization like pseudopodia, scroll-like arrangement of a lipid bilayer called myelin bodies, ballooning degeneration, swelling of mitochondria, shrunken or fragmented nucleus. Those dead cells were often in contact to pseudopodia of neighbor healthy macrophages. This suggests that dead cells were phagocytosed. The healthy macrophages also displayed intracytoplasmic vacuoles with debris suggesting phagocytosis of extracellular debris or autophagocytosis. Autophagy is a major mechanism by which macrophages eliminate intracellular pathogens or noxious particles^33^. Together, cytotoxic and structural observations indicate that Zn_0.15_Mg_0.85_O at a nontoxic dose induced death of some macrophages that are secondary phagocytyzed by the remaining healthy ones.

### Zn_0.15_Mg_0.85_O potentiates the activity of ciprofloxacin

**Figure 6:**
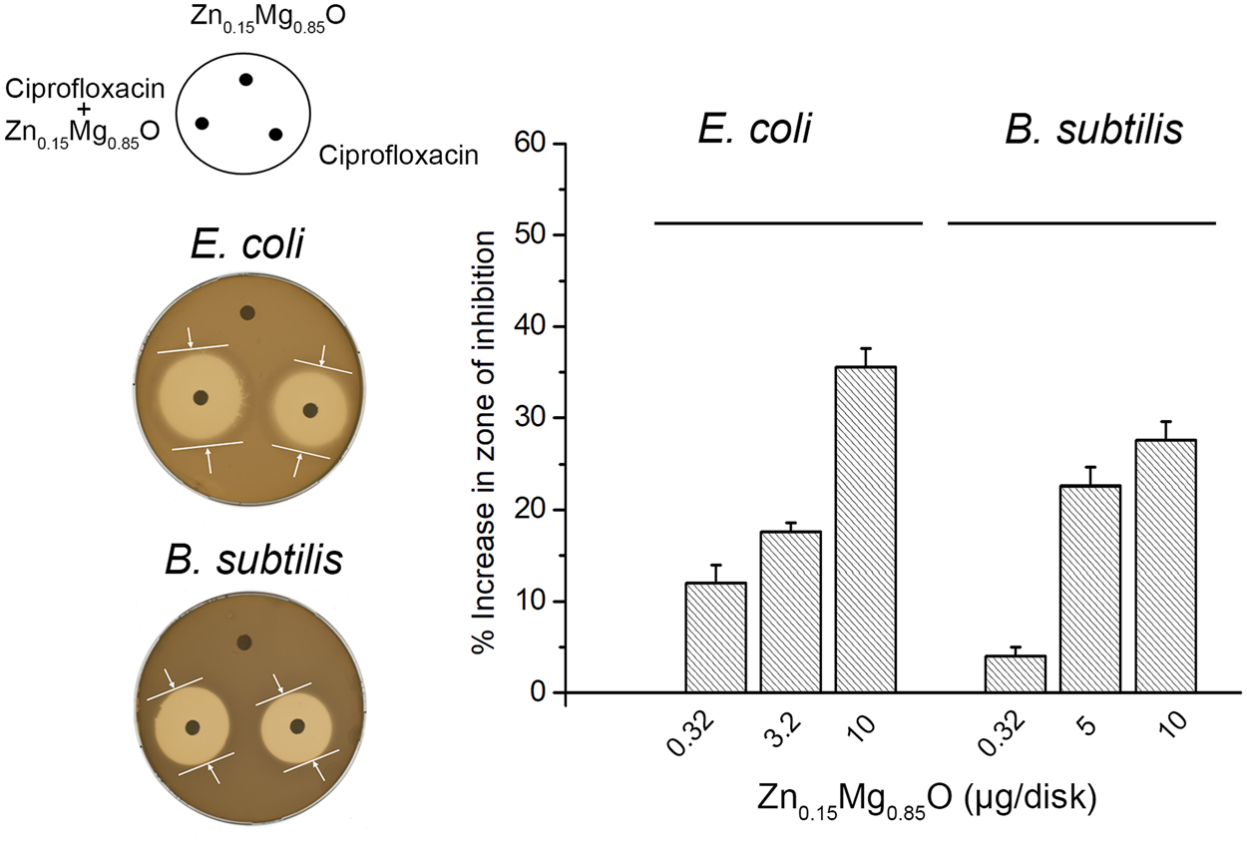
Zn_0.15_Mg_0.85_O potentiates antibiotic activity of ciprofloxacin against *B*. *subtilis* and *E*. *coli*. Paper disk diffusion assay was performed on BHI agar plates with disks loaded with 5 µg ciprofloxacin alone or co-loaded with various amount of Zn_0.15_Mg_0.85_O NPs. Disks loaded only with corresponding amounts of Zn_0.15_Mg_0.85_O NPs were tested as controls. Left panel illustrates the distribution of disks per plate and two scanned plates showing the effect of Zn_0.15_Mg_0.85_O NPs on the activity of ciprofloxacin. For both strains tested, the inhibition zone around disc co-loaded with Zn_0.15_Mg_0.85_O and ciprofloxacin was broader than that around disk loaded only with ciprofloxacin. Bars indicates the percent increase in zone of inhibition for ciprofloxacin with NPs relative to that of ciprofloxacin alone. Error bars are 95% confidence intervals.

We finally examined whether Zn_0.15_Mg_0.85_O has an additive effect when applied together with ciprofloxacin. Ciprofloxacin is a fluoroquinolone antibiotic, widely used for human and livestock treatments because of its broad-spectrum activity against both Gram (+) and Gram (−) bacteria. The contributing effect of Zn_0.15_Mg_0.85_O was tested against *B*. *subtilis* and *E*. *coli* by disk diffusion method. As shown in Fig. 6, sub-inhibitory levels of Zn_0.15_Mg_0.85_O (0.32 µg to 10 µg/disk) increased the zone of inhibition by ciprofloxacin against both bacterial strains. This implies that Zn_0.15_Mg_0.85_O has a potentiation effect on ciprofloxacin activity. However, Zn_0.15_Mg_0.85_O (up to 10 µg/disk) had no effect on the activity of penicillin and vancomycin towards *B*. *subtilis* and *E*. *coli*.

## Discussion

The innovative nanomaterials that kill multidrug-resistant bacteria and disturb antibiotic resistant biofilm are needed for industry, agriculture and healthcare. Here, we showed that (i) Zn_0.15_Mg_0.85_O killed planktonic *B*. *subtilis* cells (MIC 450 mg/L), and *E*. *coli* cells (MIC 710 mg/L), (ii) sub-inhibitory concentration of Zn_0.15_Mg_0.85_O (100 mg/L) prevented biofilm formation by NDmed strain, (iii) sub-inhibitory concentration of Zn_0.15_Mg_0.85_O (50 mg/L) modified expression of 62 membrane proteins in *B*. *subtilis* and (iv) sub-inhibitory amounts of Zn_0.15_Mg_0.85_O potentiated antibacterial efficiency of ciprofloxacin towards *B*. *subtilis* and *E*. *coli* in a dose-response manner.

Zn_0.15_Mg_0.85_O NPs aggregated in water and PBS but tended to dissolve in biological media when coated with proteins and non-ionic surfactants. MgO NPs were shown to mainly produced ROS when their electrons localized in crystal structure defects and holes, that have high oxidation and reduction energies, reduce molecular oxygen to superoxide ion (O_2_^*−^)^6^. Subsequently O_2_^*−^ can become a precursor of highly cytotoxic species as hydroxyl radicals (*OH) or singlet oxygen (1O^2^). In contrast, ZnO NPs were shown to generate mainly H_2_O_2_ and OH^34^. Hydrogen peroxide is usually generated upon water oxidation by photo-generated holes forming hydroxyl radicals. Interestingly, we observed that Zn_0.15_Mg_0.85_O NPs produced both O_2_^*−^ and H_2_O_2_ when admixed in aqueous solutions. The production of both ROSs increased with increasing NP concentration but saturated at 1 mg/mL Zn_0.15_Mg_0.85_O. Likely, NPs aggregated at high concentrations, which reduced their surface reactivity, and thus the production of ROS.

Oxidative stress in bacteria induced by ROS is considered to play a key role in molecular mechanism of metal oxide NP antibacterial activity. Our proteomic data highlight that ROS generation in combination with Zn^2+^- and Mg^2+^-ion release from Zn_0.15_Mg_0.85_O NPs triggered a broad oxidative stress response (Table 1). For instance, Spx and YraA proteins related to thiol oxidative stress, which interferes with zinc metabolism^35^ were up-regulated. Synthesis of both proteins are under the control of the regulator CzrA, which is an indicator of Zn-ions excess. This response suggests an intracellular dissolution of up-taken Zn_0.15_Mg_0.85_O. Remarkably, upregulated GcsH is involved in lipoic acid biosynthesis but also acts as an antioxidant and free-radical scavenger^36^. The exposure to NPs induced other stress response-related proteins, such as RsbW, which is involved in control of the general stress sigma factor SigB activity, and DnaK, a chaperone protein activated in response to heat shock. Several of up-regulated proteins have a function related to cation-dependent cellular processes. For instance, the phosphatase PrpC requires divalent metal cations such as Mg^2+^ or Mn^2+^ to be active. The SepF protein is a part of the divisome and recruits FtsZ to the membrane. It has been shown that Mg^2+^ impact cell division of bacilli due to its involvement in FtsZ assembly^37^.

In addition, *B*. *subtilis* recruited proteins that participate in translation and transcription cellular processes that depend on Zn and Mg availability (Table 2). The levels of at least 10 ribosomal proteins were affected by the NPs. In *B*. *subtilis*, composition of ribosomal sub-units can be modified in response to zinc availability^38^. Moreover, up-regulated RpoZ protein is part of the RNA polymerase. Structure and assembly of RNA polymerase multisubunits require Zn^2+^ while its catalytic activity is assisted by Mg^2+^ ^39^. Similarly, an addition of zinc markedly increased yields of active RNA polymerase in *Escherichia coli*^40^. Zn_0.15_Mg_0.85_O also impacted metabolic pathways. Both up-regulated proteins BglH and BglP (also named Ptv3b, Table 2) are involved in the specific carbon source utilization. In *Streptococcus pyogene*, shifts in metabolic pathways occured in response to zinc excess^41^. This further suggests that Zn_0.15_Mg_0.85_O NPs disrupt *B*. *subtilis* zinc homeostasis leading to central carbon metabolism adjustments. Together proteomic findings indicate that *B*. *subtilis* activated multiple mechanisms to recompense for the damage caused by Zn_0.15_Mg_0.85_O NPs to the cell physiology.

Proteomic data, thus, strongly suggest difficulties for bacteria to become resistant to Zn_0.15_Mg_0.85_O NPs, which makes Zn_0.15_Mg_0.85_O a promising antibacterial agent. Indeed, the multiple simultaneous mechanisms of action against bacterial cells would require multiple simultaneous gene mutations to make them resistant. Recently was shown that Gram (−) bacteria might develop resistance to silver NPs after repeated exposures^42^. The mechanism seems to involve the production of the adhesive flagellum protein flagellin, which binds and extracellularly aggregates NPs. We also observed the accumulation of NPs at the external surface of *B*. *subtils* cells (Fig. 2C). Since Zn_0.15_Mg_0.85_O NPs were of negative surface potential, their accumulation on the negatively charged bacterial surface suggests that *B*. *subtils* made efforts to sequestrate extracellularly NPs, probably to prevent their entry into the cell.

Macrophages are a canonical model of immune-competent cells that are likely to afford the first-line of defense responsible for clearing, processing and degrading foreign materials from circulation. As expected, macrophages phagocytized Zn_0.15_Mg_0.85_O NPs. Upon 24 h of incubation with macrophages, NPs were observed segregated into membrane rich region or dispersed within electron dense area. Such localization suggests that macrophages preceded their transformation as previously observed with Fe_3_O_4_ NPs^43^. Interestingly, many treated macrophage cells that showed loss of pseudopodia, swelling mitochondria or fragmented nucleus where linked to pseudopodia of neighbor healthy macrophages suggesting that damaged cells were eliminated by the secondary phagocytose. However, increasing concentration of Zn_0.15_Mg_0.85_O NPs to 1000 mg/L impeded biodegradation mechanism and led to macrophage death.

We show that sub-inhibitory amounts of Zn_0.15_Mg_0.85_O applied with ciprofloxacin had higher antibacterial efficiency compared to ciprofloxacin alone towards *E*. *coli* and *B*. *subtilis*. This finding suggests a synergistic bacterial killing that may result from the additive bactericidal activity of ROS generated by Zn_0.15_Mg_0.85_O NPs with that of ciprofloxacin, which inhibits bacterial DNA gyrase and cell division. Our proteomics data suggest that Zn_0.15_Mg_0.85_O affected bacterial physiological state, which may also increase bacterial susceptibility to antibiotics. Previously was shown that nano-ZnO enhanced activity of ciprofloxacin and ceftazidime against *A*. *baumannii* by modifying bacterial morphology from rod to cocci forms and by inducing bacterial filamentation^44^. Similarly, we observed that Zn_0.15_Mg_0.85_O NPs modified bacterial morphology and damaged cell membrane. The increased permeability of bacterial cell membrane facilitates ciprofloxacin uptake, which is expected to enhance its efficiency. Nevertheless, metal oxide NPs as well as divalent metal ions were reported to complex antibiotics and improve their antibacterial affinity^45–47^. For instance, protonated nitrogen atoms of ciprofloxacin quinolone ring may directly bind hydroxylated Zn_0.15_Mg_0.85_O NPs by ionic bonds as evidenced for some divalent metal ions by spectroscopic and X-ray analyses^48^. In addition, the oxygen from the carbonyl group of the ciprofloxacin ring was shown to bind Mg^2+^-ions forming stable complexes^49^. Such interactions between ciprofloxacin and divalent metal ions were shown to facilitate ciprofloxacin interaction with bacterial DNA^50^. Moreover, the efficiency of the Zn^2+^-ciprofloxacin complex was shown to be additionally increased by addition of H_2_O_2_^51^. To elucidate the exact mechanism of Zn_0.15_Mg_0.85_O enhancing effect on ciprofloxacin activity a deep structural-functional study remains to be done.

In conclusion, Zn_0.15_Mg_0.85_O NPs are a promising antibacterial agent as exert multiple effects on bacterial cells. The efficiency of Zn_0.15_Mg_0.85_O may be inhibited by particle aggregation in solution that reduce ROS production and metal ion release, and probably by their aggregation at the bacterial surface. The activation of multiple cellular mechanisms by Zn_0.15_Mg_0.85_O, suggests that bacteria need multiple simultaneous gene mutations to acquire resistance to mixed metal oxide NPs. We expect that further sustainable development of antibacterial metal oxide NPs will combine various doping and coating of particles to deliver safe nanomaterials that kill infection agents at high efficiency. Since effects of metal oxide NPs are additive with that of other compounds, the combination of Zn_0.15_Mg_0.85_O NPs with currently used antibiotics could be helpful to prevent new antibiotic resistance crises. In addition, different surfaces can be coated with highly stable and uniform Zn_0.15_Mg_0.85_O NPs to, which opens the way for a wide range of applications in agriculture, industry and medicine.

## Methods

### Synthesis of Zn_0.15_Mg_0.85_O NPs

Nanoparticles were prepared and characterized as previously described (see Supporting Information S1, for details)^18,19^.

### Bacterial strains, growth conditions and antibiotics

*Bacillus subtilis* 168 strain (lab’s collection), *Escherichia coli* TGI strain, and *Bacillus subtilis* NDmed strain^52^ (a kind gift from Roman Briandet) were cultivated in LB medium (10 g/liter tryptone, 5 g/liter yeast extract, 5 g/liter NaCl). Biofilm formation was studied in MSgg medium (5 mM potassium phosphate (pH 7), 100 mM MOPS (pH 7), 2 mM MgCl_2_, 700 μM CaCl_2_, 50 μM MnCl_2_, 50 μM FeCl_3_, 1 μM ZnCl_2_, 2 μM thiamine, 0.5% glycerol, 0.5% glutamate, 50 μg mL^−1^ tryptophan, 50 μg mL^−1^ phenylalanine). A Penicillin, ciprofloxacin and vancomycin were from Sigma.

### Antibacterial activity and Minimum Inhibitory Concentration (MIC) estimation

Overnight cultures of *B*. *subtilis* 168 were diluted in fresh LB medium to initial OD_600_ of 0.1 and incubated in flasks with shaking (200 rpm) at 37 °C. Zn_0.15_Mg_0.85_O NPs were added at final concentration of 0.1 or 1 mg/ml. Bacterial growth was measured by following the optical density at 600 nm. A blank containing the equivalent concentration of nanoparticles in LB medium incubated under the same conditions was used as control. After 0, 150 and 330 min of incubation with NPs, bacterial cultures were taken, successively diluted in LB medium and plated onto LB plates. Colonies forming units (CFU) were counted after incubation at 37 °C overnight. All experiments were performed in triplicate and averaged. To determine the MIC, bacterial cells (10^4^ cfu/mL) were inoculated into fresh brain heart infusion (BHI) broth in 96-microtitre plates containing varying concentrations of NPs (0.01–10 mg/L) and grown overnight at 37 °C. The wells containing no NP were positive controls while wells containing no bacteria served as a negative control. The MIC was found as a minimal concentration of NPs preventing the culture to be turbid.

### Quantitative biofilms assays

The microtiter plate assay quantitates the cells attached to the wells. Precultures of *B*. *subtilis* NDmed at OD_600_ of 1.0 were diluted in fresh LB or in MSgg to a final OD_600_ of 0.01. Samples of 125 µl of the diluted cells were inoculated in wells of a 96-well polyvinylchloride (PVC) microtiter plate (Falcon 35911). Zn_0.15_Mg_0.85_O NPs were added at final concentration of 0.1 mg/ml. Microtiter plates were incubated without agitation at 30 °C. Biofilm amount was measured by discarding the medium, rinsing the wells with phosphate buffered saline (PBS) once, and staining bound cells with a 1% crystal violet solution at room temperature for 20 min. The wells were then washed with PBS buffer three times. The dye was solubilized with acetone:ethanol 20:80, and absorbance at 595 nm was determined using a microtiter plate reader. For each experiment, background staining was corrected by subtracting the crystal violet bound to control wells. To perform pellicle assay 2 µl of bacterial culture grown at 37 °C upon agitation to an OD_600_ ~ 0.6 was added to 2 mL of MSgg (alone or with admixed NPs) in a well of 6-well microtiter plate. The plates were incubated without agitation at 30 °C for 72 h and 144 hours. Photographs were required with Samsung Galaxy smartphone. Each assay was performed at least in three independent experiments.

### Preparation of *B*. *sutilis* spores

*B*. *subtilis* 168 cells were induced to sporulate by nutrient exhaustion in Difco sporulation medium (DSM)^53^. A single colony was picked from a fresh agar plate and used to inoculate 25 ml of DSM and allowed to grow at 37 °C for 48 h. Spores and other cells/cellular debris were collected by centrifugation and washed twice with distilled water. The pellet was then resuspended in 1 ml of distilled water. A heat treatment (20 min at 80 °C) was applied to eliminate vegetative cells. Zn_0.15_Mg_0.85_O NPs were added to spores suspension at final concentration of 1 or 5 mg/ml. After 24 h of incubation, the number of CFU in the presence and in the absence of NPs was determined by plating dilutions on agar plates.

### Disk diffusion assays

One mL of exponentially growing cells (OD_600_ = 0.8–1) of the strain being tested was spread on the Petri plates containing BHI agar medium. The plates were allowed to dry briefly in a laminar flow before 6.6 mm filter paper disks (Whatman) containing the antibiotics and/or nanoparticles (20 µL volume) were placed on the plates (ciprofloxacin 250 µg/mL, penicillin 16 mg/mL, vancomycin 1 µg/mL). The plates were incubated at 37 °C overnight and the zones of inhibition were measured. The values in Fig. 6 are an average of three independent experiments.

### Cell culture and MTT test

The immortalized murine peritoneal macrophage cell line RAW 264.7 from the American Type Culture Collection was utilized to determine cytotoxic effect of Zn_0.15_Mg_0.85_O. Macrophages were grown in complete Dulbecco’s Modified Eagle Medium (DMEM) medium (Eurobio, France) supplemented with 2 mM glutamine, antibiotics (100 U/ml penicillin A and 100 U/ml streptomycin) and 10% heat-inactivated fetal bovine serum and maintained in a humidified incubator at 37 °C under 5% CO_2_. Macrophages were plated at a density of 30,000 cells per well on 96-well plates. After 24 h, cell medium was exchanged and various concentrations of Zn_0.15_Mg_0.85_O were loaded per well and incubated for 1 h. Afterwards, a freshly prepared 3-(4,5-dimethylthiazol-2-yl)-2,5-diphenyltetrazolium bromide (MTT) at final concentration of 0.8 mg/ml was added and incubated for a further 1 h. Then, the cell layer was dried and MTT formazan produced by conversion of the water soluble MTT was suspended in 100 μl of dimethyl sulfoxide. Cell survival was quantified by measuring absorbance values assessed at 560 nm and corrected for a background signal by subtracting the signal measured at 670 nm. Cell survival was expressed as % of cells treated only with water (mock).

### Quantitation of ROS

The Amplex red assay was used to quantify H_2_O_2_ production. Different amount of Zn_0.15_Mg_0.85_O NPs were admixed with various solutions and incubated during 1 hour in the dark. The suspensions (80 µL) were transferred in a 96-wells plate that contained 20 µl of enzymatic mix (1 µl 10-Acetyl-3,7-dihydroxyphenoxazine (ADHP) reagent, 1 µl horseradish peroxidase and 18 µl assay buffer) in each well. Resorufin fluorescence was measured using a spectrofluorometer (Tecan infinite M200PRO) with excitation and emission wavelengths of 530 and 590 nm, respectively. H_2_O_2_ calibration was obtained using H_2_O_2_ standard solutions ranging from 100 to 1500 nM. Each experiment was performed in triplicate and repeated at least twice.

The production of superoxide radical ion (O_2_^*−^) was evaluated by measuring the adsorption of XTT (2,3-bis (2-methoxy-4-nitro-5-sulfophenyl)-2H-tetrazolium-5-carboxanilide, Sigma). XTT absorbs light at 470 nm when reduced by O_2_^*−^ but not in its oxidized form. XTT dissolved in PBS, pH 7 (0.4 mM). Different concentrations of NPs were mixed with XTT at 0.2 mM and incubated in dark for 5 h. Afterwards, the mixtures were filtered through 0.22 µm syringe filters (Millex) to remove aggregated NPs. The changes in absorbance at 470 nm were monitored using an UV-Vis spectrophotometer.

### Cell morphology observation with TEM

TEM analysis were performed to visualize the Zn_0.15_Mg_0.85_O effects on morphology of *B*. *subtilis* cells using Hitachi HT7700 electron microscope operated at 80 kV (Elexience, France). Bacterial cells at OD_600_ = 1 were incubated with 0.1 mg/ml Zn_0.15_Mg_0.75_O for 1 hour. Cultured cells were fixed with 2% glutaraldehyde in 0.1 M sodium cacodylate buffer (pH 7.2) at room temperature for 1 h. Samples were then contrasted with 0.5% Oolong Tea Extract in sodium cacodylate buffer and post-fixed with 1% osmium tetroxide containing 1.5% potassium cyanoferrate, gradually dehydrated in ethanol (30 to 100%), and substituted gradually in a mixture of propylene oxide-epon and embedded in Epon (Delta Microscopy, Labège, France). Thin sections (70 nm) were collected onto 200-mesh copper grids, and counterstained with lead citrate to allow TEM visualization. Digital images were acquired using a charge-coupled device camera system (AMT).

### DLS

The Z-potential of the NP colloidal solutions was measured using a Zetasizer Nano ZS90 (Malvern, UK). The results of zeta potential are presented as the average value of three measurements ± SD. Particle size measurements were performed on a Zetasizer Nano-S (Malvern, UK) at 20 °C using a helium-neon laser wavelength of 633 nm and detection angle of 173°. A total of 10 scans with an overall duration of 5 min were performed for each sample. The results were presented as a size distribution.

### FACS analysis

Macrophage cells were incubated at 37 °C in complete medium with various concertation of Zn_0.15_Mg_0.85_O for 24 h. Cell death was quantified by acridine orange staining followed by cytometry analysis (BD LSRFortessa, BD Bioscience) with the 488-nm laser line and the FITC (530/30) channel. For this, cells were collected by centrifugation, washed two times in PBS and resuspended in MEM containing 0.1 µg/mL acridine orange dye. After incubated for 10 min in the dark, stained cells were collected, washed two times with PBS and fixed with 3.5% paraformaldehyde in PBS for 30 min. The fixed cells were collected and resuspended in PBS for analysis.

### Proteomics analysis

Membrane fraction preparation and proteomics analysis were performed as described in the Supplementary Information S4.

## Electronic supplementary material

Supplementary Information

Dataset 1
