# Supplementary material for: Exploring multiple effects of Zn0.15Mg0.85O nanoparticles on Bacillus subtilis and macrophages
Source: Sci Rep. 2018 Aug 16;8:12276. doi: 10.1038/s41598-018-30719-9 (PMC6095908; doi:10.1038/s41598-018-30719-9)
Supplement: Supplementary file 1 — Supplementary Information [file 41598_2018_30719_MOESM1_ESM.docx]

# Exploring multiple effects of Zn_0.15_Mg_0.85_O nanoparticules on *Bacillus subtilis* and macrophages

# Sandrine Auger^1^, Céline Henry^2^, Christine Péchoux^3^, Sneha Suman^4,5^, Nathalie Lejal^6^, Nicolas Bertho^6^, Thibaut Larcher^7^, Slavica Stankic^4^, Jasmina Vidic^1,6*^

^1^ Micalis Institute, INRA, AgroParisTech, Université Paris-Saclay, 78350 Jouy-en-Josas, France.

^2^ Micalis Institute, PAPPSO, INRA, AgroParisTech, Université Paris-Saclay, 78350 Jouy-en-Josas, France.

^3^ Université Paris-Saclay, Génétique Animale et Biologie Intégrative, UMR1313, INRA, France.

^4^ Sorbonne Université, UPMC Paris 06, CNRS-UMR 7588, Institut des NanoSciences de Paris, France.

^5^ Department of Chemical and Biomolecular Engineering, Whiting School of Engineering, Johns Hopkins University, Baltimore, USA.

^6^ Université Paris-Saclay, Virologie et Immunologie Moléculaires, UR892, INRA, Jouy-en-Josas, France.

^7^ INRA, UMR0703 APEX, Oniris, Nantes, France.

To whom correspondence should be addressed. E-mail: [jasmina.vidic@inra.fr](mailto:jasmina.vidic@inra.fr)

# Synthesis and Characterization of Zn_0.15_Mg_0.85_O NP. Zn_0.15_Mg_0.85_O nanocubes, with average size of 4 nm, were obtained via chemical vapor synthesis (CVS). Details of this technique are given elsewhere^1^. High purity Zn and Mg pieces supplied by Advent Research Materials Ltd were used as starting material. The concentrations of Zn^2+^ and Mg^2+^ were determined by atomic absorption spectroscopy (PerkinElmer model 2280). The nanoparticle surface was dehydroxilated in a thermally activated process that comprises annealing of the powder at 1270 K under high vacuum conditions (P<10^-5^ mbar). Transmission Electron Microscopy (TEM) measurements were achieved by using a LaB6 JEOL JEM 2100 (JEOL, Japan) field emission transmission electron microscope operated at 200 kV and with 0.18 nm resolution. The representative TEM image of Zn_0.15_Mg_0.85_O NPs is shown in Fig. S-1.

#
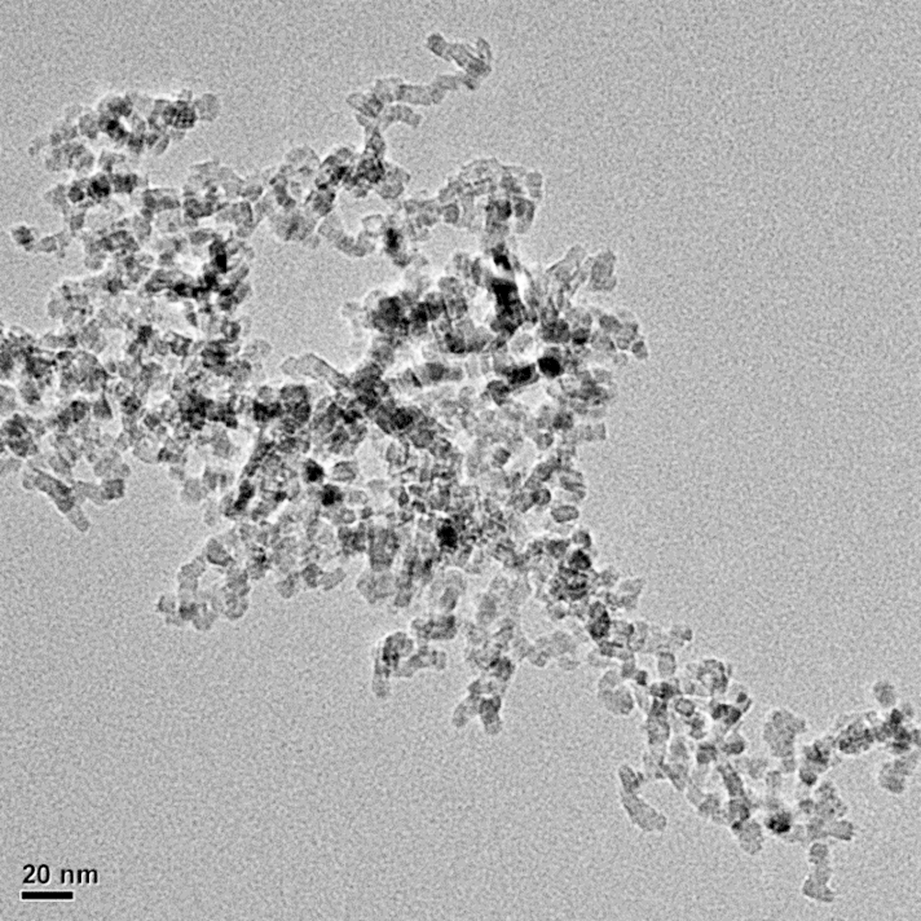


# Fig. S-1. TEM image of Zn_0.15_Mg_0.85_O nanoparticles obtained via CVS method illustrates regular cubic crystal form of particles. Powder was kept at P < 10^−5^ mbar after the synthesis.

1. **Antibacterial activity of Zn_0.15_Mg_0.85_O on *Escherichia coli*.** The antibacterial efficiency of Zn_0.15_Mg_0.85_O against *E. coli* was tested by measuring the optical density of bacteria incubated with NPs over time, and by the colony counting method. As shown in Fig. S-2A, the growth of *E. coli* decreased significantly with the increasing concentration of NPs. Estimated cellular viability was about 2.5-log reduced as compared to the initial concentration of cells (10^8^ CFU/ml) upon incubation with 1 mg/mL Zn_0.15_Mg_0.85_O NPs after 330 min (Fig. S-2B). The minimal inhibitory concentration (MIC) value of Zn_0.15_Mg_0.85_O NPs against *E. coli* evaluated by broth microdilution method was 750 ppm.

#
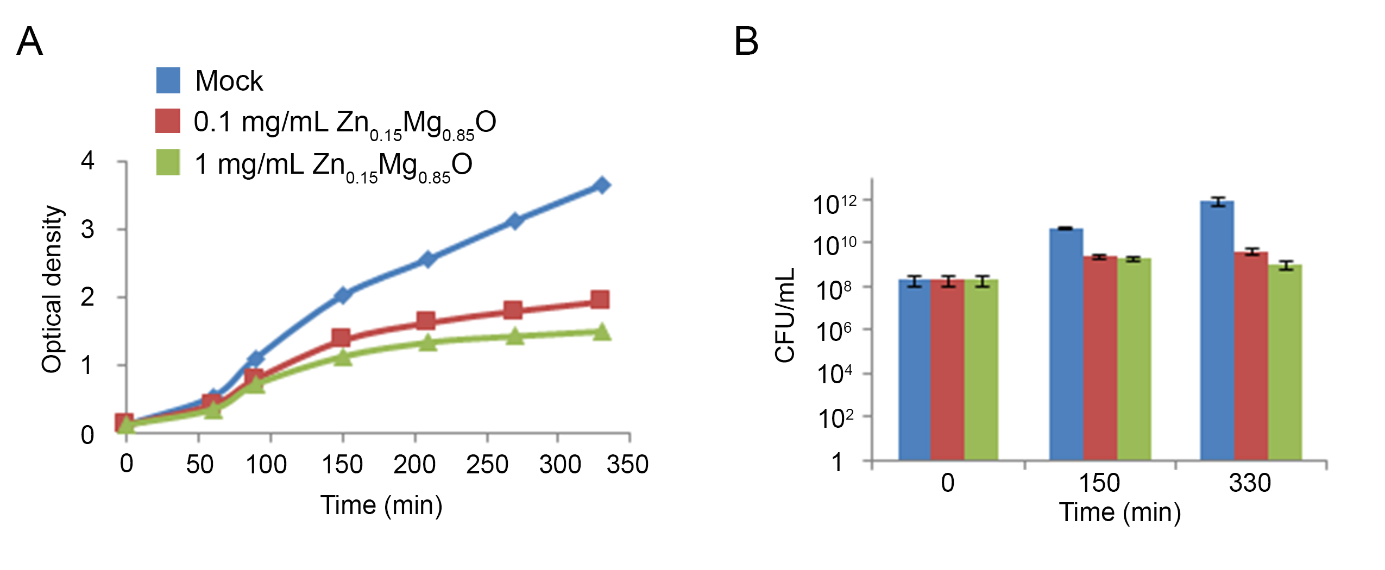


# Sample preparation for proteomics. *B. subtilis* was cultivated in 40 mL of LB medium to rich DO_600_=0.9. Then, 20 mL were incubated with 20 µL of 50 mM Zn_0.15_Mg_0.85_O NPs (to obtain 0.05 mg/mL final concentration of NPs), while 20 ml were incubated with 20 µL of water (control) for 1 h. Control bacteria were incubated in LB medium without NPs. Cells were harvested by centrifugation at 2,000 g, washed with ice-cold water and resuspended in an equal volume of ice-cold lysis buffer, 50 mm Tris/HCl pH 7.5, 1 mm EDTA, 250 mm sorbitol and the Complete protease inhibitor cocktail (Roche). Cells were then disrupted by 3 sonication cycles of 1 min of sonication/1 min of cooling on ice. Samples were first centrifuged at 5,000 g for 10 min at 4 °C to remove unbroken cells and cell walls and second at 40,000 g for 40 min at 4 °C. The pellet obtained after the second ultracentrifugation step, enriched in membranes, was resuspended in the lysis buffer and stored in aliquots at −80 °C. The total protein concentration of the membrane preparation was determined using the BCA reagent (Pierce, Brebieres, France) with bovine serum albumin (BSA) as a standard. Protein profiles were controlled by one-dimensional SDS-PAGE analysis. For a high-resolution mass spectroscopic analysis, in-gel digestion of proteins was performed after short migration of proteins (10 mg protein total). Statistical analysis was performed correlating the identified peptides with the reference ones following the workflow from the Fig S-3.

# In-gel digestion of the proteins was performed on bands excised from one-dimensional SDS-PAGE. Each lane of short migration was cut and washed for 15 min with an acetonitrile/100 mM ammonium bicarbonate mixture (1:1). Digestion was performed in 50 mM ammonium bicarbonate pH 8.0 and the quantity of modified trypsin (Promega, sequencing grade) was 0.1 μg per sample. Digestion was achieved for 6 h at 37°C. The supernatant was conserved. Peptides were extracted by 5% formic acid in water/acetonitrile (v/v). Supernatant and extracted tryptic peptides were dried and resuspended in 50 µL of of nano HPLC buffer containing 0.1% (v/v) formic acid and 2% (v/v) acetonitrile.

**Liquid Chromatography – Mass Spectrometry.** Mass spectrometry was performed on the PAPPSO platform^2^. An Orbitrap Fusion^TM^ Lumos ^TM^ Tribrid ^TM^ (Thermo Fisher Scientific) coupled to an UltiMate™ 3000 RSLCnano System (Thermo Fisher Scientific) was used. A 4 μl sample was loaded at 20 μl/min on a precolumn (µ-Precolumn, 300 µm i.d x 5 mm, C18 PepMap100, 5 µm, 100 Å, Thermo Fisher) and washed with loading buffer. After 3 min, the precolumn cartridge was connected to the separating column (Acclaim PepMap®, 75 μm x 500 mm, C18, 3 μm, 100 Å, Thermo Fisher). Buffer A consisted of 0.1 % formic acid in 2 % acetonitrile and buffer B of 0.1 % formic acid in 80 % acetonitrile. The peptide separation analysis was achieved at 300 nl/min with a linear gradient from 1 to 35 % buffer B for 145 min and 35 % to 45 % for 25 min. One run took 210 minutes including the regeneration step at 98 % buffer B. Ionization (1.6 kV ionization potential) and capillary transfer (275 °C) were performed with a liquid junction and a capillary probe (SilicaTip™ Emitter, 10 μm, New Objective). Peptide ions were analyzed using Xcalibur 3.1.66.10. In CID mode the machine settings were as follows: (i) full MS scan in Orbitrap (scan range [m/z] = 400–1500) with a resolution of 120 000 (AGC target = 4.0 x 10^5^, max. injection time of 50 ms, data type = profile). Analyzed charge states were set to 2-5, the dynamic exclusion to 60 s and the intensity threshold was fixed at 1 x 10^4^, and (ii) MS/MS using CID (35 % collision energy) in trap (AGC target = 5.0 x 10^3^ max. injection time = 100 ms).

The *Bacillus subtilis* strain 168 database (version 12/13/2016, 4185 entries) was searched by the X!TandemPipeline (open source software developed by PAPPSO, version 3.4.3, ^3^. The proteins identification was run with a precursor mass tolerance of 10 ppm and a fragment mass tolerance of 0.5 Da. Enzymatic cleavage rules were set to trypsin digestion (“after Arg and Lys, unless Pro follows directly after”) and no semi-enzymatic cleavage rules were allowed. The fix modification was set to cysteine carbamidomethylation and methionine oxidation was considered as a potential modification. The identified proteins were filtered as follows: (i) peptide E‑value < 0.01 with a minimum of 2 peptides per protein and (ii) protein E-value of < 10^-4^.

Peptide quantities of the proteome were analyzed by spectral counting (SC) and eXtracted Ion Current (XIC). SC takes into account the number of assigned spectra for each protein and is correlated to relative protein abundance. For the quantification of peptides by (XIC), MassChroQ “Black Caiman” version 0.3.7 ^46^ was used. The range for peak detection was set to 10 ppm with a detection threshold ranging from 30000 to 50000. All peptide intensities were log_10_‑transformed for the following data treatments. The analysis using XIC requires the alignment of retention times, intensity normalization and log-transformation of values. Protein abundances are calculated as the sum of peptide intensities, which allows for analysis of quantitative variations^4^. Proteins with less than two peptides were removed. Peptides with a variation ratio < 1.5 were eliminated, as well as peptides with a standard deviation from the retention time of 20 s and higher. During the XIC analysis, the following data was eliminated additionally: peptides with a peak width higher than 100 s, peptides absent in more than 5 % of samples and proteins quantified by a small number of peptides-modification-charge combination. The data set was normalized based on the median RT and missing peptide intensities and protein abundances were imputated. The significance of variation was determined by an ANOVA (analysis of variance). The p-values obtained from ANOVA were considered significant below a value respectively for the SC and the XIC: 0.05 and of 0.01.

#
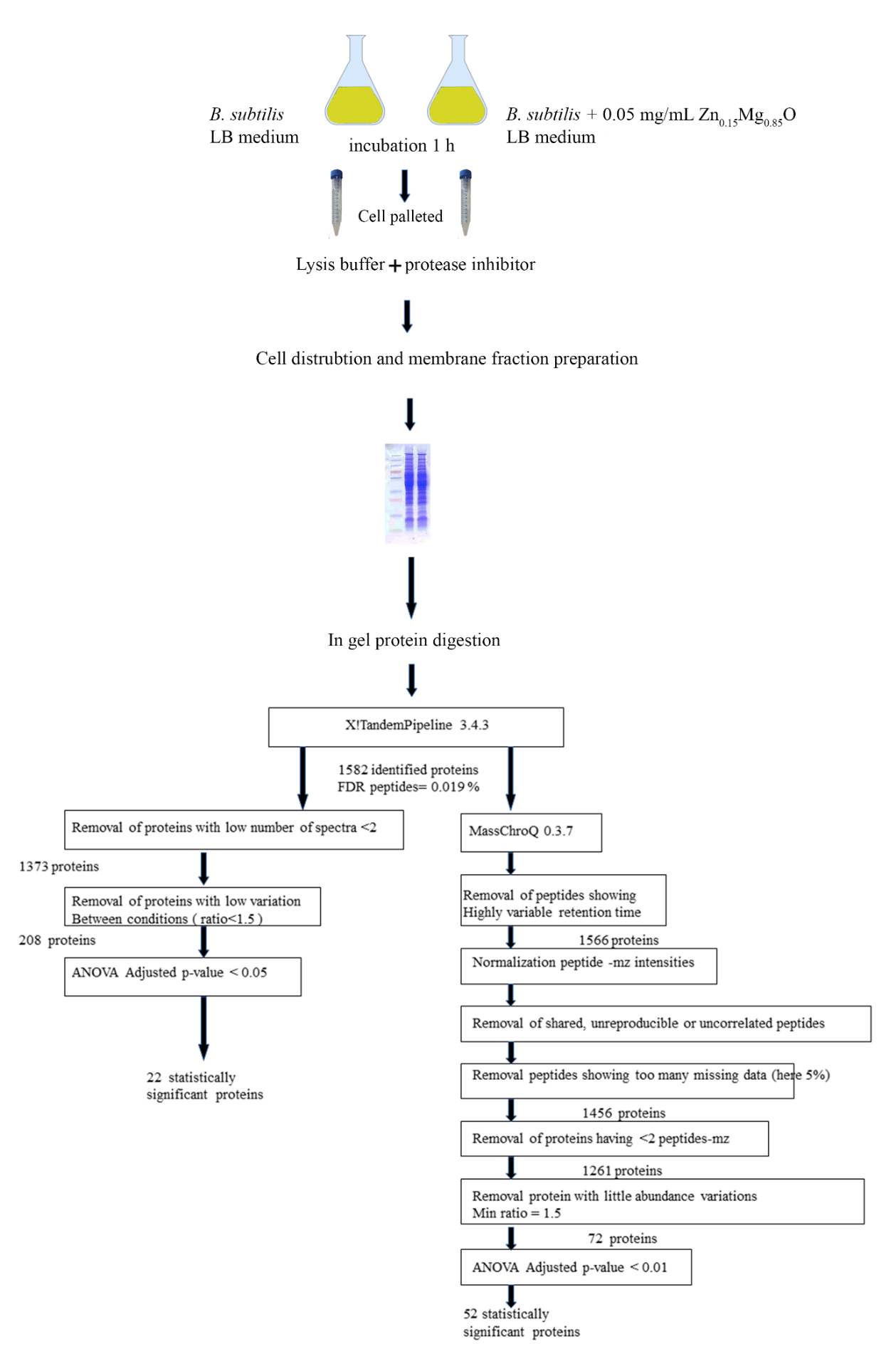


# Fig. S-3. General workflow of membrane fraction preparations for proteomics and the computing and statistical analysis of proteomic data.

References:

1 Stankic, S. *et al.* Size‐Dependent Optical Properties of MgO Nanocubes. *Angewandte Chemie International Edition* **44**, 4917-4920 (2005).

2 <http://pappso.inra.fr/> (2018)

3 <http://pappso.inra.fr/bioinfo/xtandempipeline/> (2018)

4 Blein-Nicolas, M. & Zivy, M. Thousand and one ways to quantify and compare protein abundances in label-free bottom-up proteomics. *Biochim Biophys Acta* **1864**, 883-895 (2016).
